# Improved clinical efficacy of melasma with combination therapy of intense pulsed light and tranexamic acid: A retrospective analysis

**DOI:** 10.1097/MD.0000000000048042

**Published:** 2026-03-13

**Authors:** Rongli Yang, Yan Zhao, Jintian Luo, Chang Liu, Yiyang Bai, Shaoli Cheng, Xin Mu

**Affiliations:** a Department of Dermatology, The First Affiliated Hospital of Xi’an Jiaotong University, Xi’an, Shaanxi, China; b Zhongshan School of Medicine, Sun Yat-Sen University, Guangzhou, Guangdong, China; c Department of Dermatology, Weinan Central Hospital, Weinan, Shaanxi, China; d Basic Medical Experiment Teaching Center, Health Science Center, Xi’an Jiaotong University, Xi’an, Shaanxi, China.

**Keywords:** effectiveness, intense pulsed light (IPL) therapy, melasma, patient satisfaction, recurrence, safety, tranexamic acid (TA) microneedling

## Abstract

This study aimed to evaluate the efficacy and safety of intense pulsed light (IPL) therapy as a standalone treatment versus IPL therapy combined with tranexamic acid (TA) microneedling for the management of melasma. Clinical data of 29 patients with melasma were reviewed retrospectively. Patients were divided into 2 groups based on their treatment regimens: the IPL group, which received 3 sessions of IPL therapy, and the IPL + TA group, which underwent IPL therapy immediately followed by TA microneedling. Patients were comprehensively analyzed before treatment, as well as one month and 3 months after the final treatment, utilizing the VISIA skin imaging system, Melasma Area and Severity Index (MASI) scoring, patient satisfaction surveys, recurrence evaluations, and assessments of tolerability and adverse reactions. Both groups exhibited notable improvements in VISIA results and MASI scores post-treatment, with no statistically significant differences between the 2 groups. However, the IPL + TA group reported higher patient satisfaction rates and a lower recurrence rate compared to the IPL group. Both treatments were well-tolerated, with no severe adverse reactions reported. Both IPL therapy alone and IPL therapy combined with TA microneedling have demonstrated efficacy and safety in the treatment for melasma. However, the combination therapy may offer greater patient satisfaction and a reduced risk of recurrence.

## 1. Introduction

Melasma is a chronic and acquired skin condition characterized by symmetrical, variably pigmented patches with fuzzy boundaries, ranging from light to dark brown in color, and typically appearing on the cheeks, forehead, and mandible. This disorder is particularly prevalent among individuals with Fitzpatrick skin phototypes III and IV, with an incidence rate reaching up to 30% among Asian women of childbearing age.^[1]^ In recent years, there has been a concerning trend of melasma occurring at a younger age. Unfortunately, clinical treatments for melasma often yield unsatisfactory outcomes and are susceptible to recurrence, leading not only to facial disfigurement but also to significant negative effects on patients’ physical and mental health, ultimately resulting in a substantial decrease in their quality of life.

Melasma can be categorized into 2 types based on the presence of vascular involvement: the simple melanized type and the melanized with vascularized type. The onset of melasma is attributed to a complex interplay of multitude of factors, including ultraviolet radiation exposure, genetic predisposition, pregnancy, endocrine abnormalities, and hormonal dysfunction. These factors collectively contribute to an increase in melanin synthesis, vascular proliferation within the affected areas, accompanied by inflammatory reactions, compromised skin barrier function, and accelerated photoaging.^[2]^

Currently, the management of melasma primarily relies on comprehensive strategies, including topical medications, oral drugs, and phototherapy. However, topical agents like hydroquinone have raised concerns due to their uncertain efficacy and skin allergies.^[3]^ Oral tranexamic acid (TA) has shown definite efficacy in melasma treatment by inhibiting tyrosinase activity to reduce melanin synthesis and inhibiting vascular proliferation. Nevertheless, potential risks associated with long-term oral use, such as reduced menstruation and thrombus formation, limit its clinical application.^[4]^ Reports also indicate that intense pulsed light (IPL) or Q-switched lasers exhibit some therapeutic effect on melasma by accelerating pigment metabolism, yet they fail to reduce melanin production, and their solitary use often results in recurrence.^[5]^

Given the diversity of treatment methods for melasma, the unpredictability of their efficacy, and the clinical challenges of recurrence or even worsening, novel treatment approaches are being actively pursued. IPL, a broad-spectrum light with wavelengths ranging from 500 to 1200 nm, can destroy pigmentation in melasma through selective photothermolysis and seal blood vessels. Although it accelerates the resolution of pigmented spots, it cannot prevent recurrence.^[6]^ Microneedling, a technique involving the puncturing the epidermis with fine needles to create microchannels, facilitates the absorption of drugs through the skin, allowing them to directly act on damaged tissue cells and thereby enhancing drug efficacy. Previous studies have demonstrated that microneedling-assisted delivery of TA is an effective melasma treatment melasma.^[7]^ This approach circumvents adverse reactions associated with oral medications and overcome the limitation of topical agents not reaching the affected areas, gradually emerging as a promising new option for melasma treatment.^[7]^ The efficacy of combining IPL with microneedling-assisted delivery of TA for melasma treatment has not yet been clearly reported. Therefore, we designed this study to evaluate the efficacy and safety of this combined treatment approach. This research aims to provide a novel and more effective treatment option for patients with melasma.

## 2. Materials and methods

### 2.1. Patients

A retrospective analysis was performed on the clinical data of 29 melasma patients admitted to the dermatology outpatient clinic of the First Affiliated Hospital of Xi’an Jiaotong University between January 2023 and January 2024. Prior to participation, all patients were fully informed about the study’s content and voluntarily provided their informed consent by signing the consent forms. The study was approved by the Ethics Committee of The First Affiliated Hospital of Xi’an Jiaotong University (no. XJTU1AF2025LSYY-372).

Inclusion criteria: Age ranging from 18 to 60 years; Melasma confirmed through a joint assessment of 2 experienced dermatologists, Wood lamp examination, and VISIA skin analysis; Completion of informed consent forms and follow-up by all patients.

Exclusion criteria: Incomplete clinical data, including missing photographs or lack of follow-up information; Use of topical bleaching agents (e.g., hydroquinone, tretinoin, alpha-hydroxy acids, etc) within the past 3 months; Receipt of laser therapy or chemical peeling within the last 6 months; Current use of hormonal contraceptives, hormone replacement therapy, or oral TA; Presence of vitiligo, dermatitis, eczema, active infectious foci, poor wound healing, keloids, malignant tumors, or immunodeficiency; Sun exposure within the past month; and Pregnancy or lactation.

### 2.2. Treatment protocol

Patients were divided into 2 groups according to their assigned treatment plans: the IPL group (n = 14) and the IPL + TA group (n = 15). Participants in the IPL group underwent a course of 3 IPL treatments, administered at monthly intervals over a span of 3 months. Those in the IPL + TA group received microneedling-assisted TA administration immediately following each IPL session, also adhering to a monthly schedule for a total of 3 treatments. Prior to the commencement of treatment, as well as one month and 3 months after the final treatment, facial images were captured at 45-degree angles from both left and right profiles, along with a frontal view, all under consistent lighting conditions and angles using VISIA skin image analysis system (manufactured by Canfield Imaging Systems, Fairfield). For the IPL group, treatments were performed using the AOPT M22 device (Lumenis Limited, Yokneam Industrial Park, Hakidma Street, Israel). A uniform layer of chilled optical coupling gel was applied to the facial area, and protective eyewear was worn throughout the procedure. Treatment parameters were carefully adjusted according to individual skin tones and lesion characteristics before initiating the IPL treatment. The specific IPL treatment parameters were as follows: an initial pass using the M22 640 filter (wavelength range: 640–1200 nm) with a triplet of pulses, a fluence ranging from 13 to 17 J/cm^2^, a pulse duration of 5.0 to 6.0 ms, and a pulse delay of 30 to 40 ms; this was followed by the M22 590 filter (wavelength range: 590–1200 nm) with a doublet of pulses, a fluence of 13 to 16 J/cm^2^, a pulse duration of 3.0 to 5.0 ms, and a pulse delay of 20 to 40 ms. The desired endpoint of the IPL treatment was marked by a slight darkening of pigmentation or the emergence of mild, transient erythema around the melasma lesions.

For the IPL + TA group, immediately after the conclusion of the IPL procedure, the facial skin underwent thorough cleansing and disinfection. Microneedling (Derma roller, Suzhou Xiunuo Photoelectric Technology Co., Ltd., suzhou, Chnia) was then employed to administer TA (consisting of 4 mL of a 5% TA essence solution and 0.3 grams glutathione crystal powder, Shanghai Uface Biotechnology Co., Ltd., Shanghai, Chnia) into the skin. The microneedling device, featuring 192 micro-needles with a length of 0.5 mm and a tip diameter of 200 μm, was gently rolled over the designated treatment areas on the chin, cheeks, nose, and forehead – exerting a controlled amount of pressure. The rolling motion was maintained until the skin exhibited a slight redness and swelling, accompanied by minimal punctate bleeding. Subsequently, the TA solution was applied, and the rolling resumed while the practitioner gently massaged the skin to ensure full absorption of the solution. Upon completion of each treatment session for both groups, patients were provided with a chilled, sterile facial mask to apply as a cold compress for 20 to 30 minutes, aiding in the mitigation of any potential thermal injury. Following the removal of the mask, a mild moisturizer was carefully applied to the treated regions. Patients who underwent microneedling were advised to refrain from exposing their faces to water for one day. Throughout the treatment duration and thereafter, all patients were strictly instructed to practice adequate sun protection measures, including the application of broad-spectrum sunscreen when venturing outdoors.

Evaluations were scheduled prior to treatment initiation, as well as one month and 3 months after the final treatment session.

### 2.3. Evaluation of clinical effectiveness

#### 2.3.1. Assessment with VISIA

Utilizing the VISIA Facial Skin Imaging System, we captured images from 3 distinct angles: frontal, 45° left lateral, and 45° right lateral. These images were specifically analyzed for 4 key indicators: Spots, UV Spots, Brown Spots, and Red Areas. By comparing the score values obtained for these indicators prior to treatment, as well as one month and 3 months after the final treatment session, we evaluated the improvement in pigmentation and vasculature related to melasma.

#### 2.3.2. MASI score assessment

Utilizing the Melasma Area and Severity Index (MASI),^[8]^ 2 dermatologists independently evaluated a randomly ordered set of melasma photographs and computed the average score. The MASI score was determined by evaluating the hyperpigmented regions of the face, which were categorized into 4 areas: forehead (F), right malar region (MR), left malar region (ML), and chin (C). Each of these zones assigned respective weights of 30%, 30%, 30%, and 10%. The degree of pigmentation within these regions was then scored (A) based on the percentage of affected area: 1 point for <10%, incrementing to 6 points for 90 to 100% coverage. Additionally, pigment darkness (D) and homogeneity (H) were rated on a 0 to 4 scale: 0 for none, 1 for mild, 2 for moderate, 3 for pronounced, and 4 for maximal. The final MASI score was calculated using the formula: MASI score = F [0.3A(D + H)] + MR [0.3A(D + H)] + ML [0.3A(D + H)] + C [0.1A(D + H)]. The score ranged from a minimum of 0 to a maximum of 48 (Table 1).

**Table 1:** Melasma area and severity index.

Clinical index		Score						
		0	1	2	3	4	5	6
Order of severity	Darkness of pigment: D	Absent	Slight	Mild	Marked	Maximum	–	–
	Homogeneity:H	Absent	Slight	Mild	Marked	Maximum	–	–
Area:A	%	0	<10	10–29	30–49	50–69	70–89	>90

#### 2.3.3. Overall patient satisfaction survey

During the follow-up period, patients self-reported their improvement in melasma treatment based on 4 key aspects: color intensity, lesion size, skin radiance, and overall satisfaction. They rated each aspect on a 0 to 100 scale: below 60 meant dissatisfaction, 60 to 70 was neutrality, 71 to 80 was satisfaction, and above 80 was high satisfaction. A higher score was indicative of a greater level of satisfaction. The satisfaction rate was calculated using the formula: total number of highly satisfied and satisfied patients/total number of patients × 100%.

#### 2.3.4. Recurrence evaluation

Recurrence was evaluated at 3 months after treatments, and defined as an increase of MASI score exceeding 50% compared with the one-month post-treatment score, and/or the observation of larged discolored area or new melasma. Total recurrence rate was determined as the percentage of affected patients.

#### 2.3.5. Tolerability and adverse reactions assessment

Throughout the entire treatment duration and during the one-month and three-month follow-up periods, patients were closely monitored for any adverse reactions. A specialized dermatologist recorded the occurrence of symptoms such as pain, erythema, blistering, exudation, desquamation, burning sensation, tightness, pruritus, and menstrual flow reduction.

### 2.4. Statistical analysis methods

The research data were analyzed statistically using SPSS 25.0 software. Normally distributed measurement data were presented as the mean ± standard deviation (x ± s). Between-group comparisons were performed using an independent sample t-test, while within-group pre- and post-treatment comparisons were analyzed using one-way ANOVA. For non-normally distributed data, the Mann–Whitney test was employed for two-group comparisons, and the Kruskal–Wallis test was used for multiple-group comparisons. Count data were presented as percentages (%), and Fisher exact test was applied for intergroup comparisons. A *P*-value <.05 was considered statistically significant.

## 3. Results

### 3.1. General data

Among the 29 patients diagnosed with melasma, 14 were assigned to the IPL group (n = 14), while 15 were assigned to the IPL + TA group (n = 15). The IPL group comprised 1 male and 13 females, with ages ranging from 28 to 50 years and a mean age of 38.6 ± 6.2 years. According to the Fitzpatrick skin phototype classification, one patient was classified as phototype III and 13 as phototype IV. The duration of their melasma ranged from 2 to 6 years, averaging 4 ± 1 years. Clinically, 10 cases were categorized as melanized type (M type) and 4 as melanized with vascularized type (M + V type). Among of them, 2 patients had concurrent seborrheic keratosis and 2 had freckles.

In the IPL + TA group, the patient population comprised 1 male and 14 females, aged between 28 and 52 years with a mean age of 38.3 ± 6.2 years. Based on the Fitzpatrick skin phototype classification, 1 patient was phototype III and 14 were phototype IV. The duration of their melasma varied from 3 to 6 years, with an average duration of 4 ± 1 years. Clinically, 11 cases were classified as melanized type (M type) and 4 as melanized with vascularized type (M + V type). Furthermore, 2 patients presented with concurrent seborrheic keratosis and 3 with freckles.

A comparative between the 2 groups revealed no statistically significant differences in age, disease duration, or clinical classification (all *P* >.05), confirming their comparability. These findings are summarized in Table 2.

**Table 2:** Comparison of general characteristics between the 2 patient groups.

Group	Number	Sex (F/M, n)	Age (yr)	Duration (yr)	Fitzpatrick phototype (Ⅲ/Ⅳ, n)	Clinical type (M/ M + V, n)
IPL	14	13/1	38.6 ± 6.2	4 ± 1	1/13	10/4
IPL + TA	15	14/1	38.2 ± 6.2	4 ± 1	1/14	11/4
χ^2^/*F* value		*F*	χ^2^	χ^2^	*F*	*F*
*P* value		>.99	.89	.54	>.99	.69

IPL = intense pulsed light, TA = tranexamic acid.

### 3.2. VISIA assessment results

**Figure 1.**
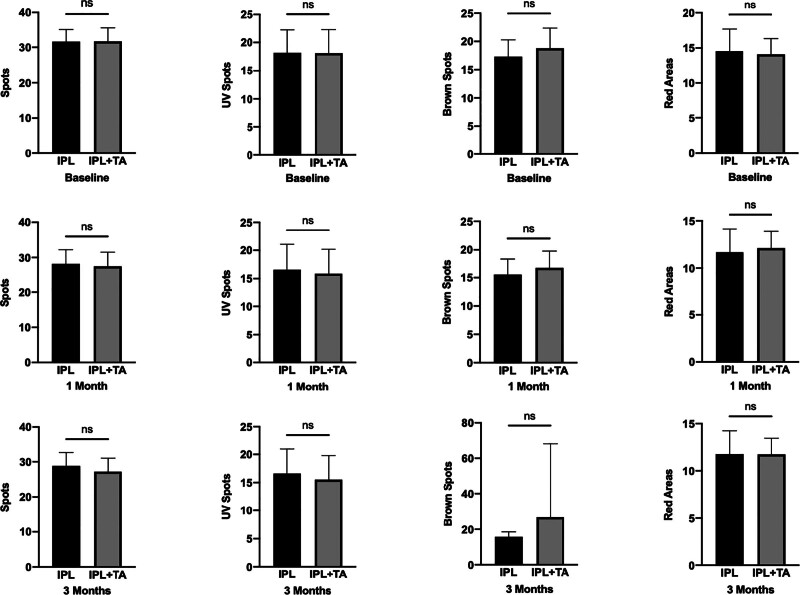
Comparison of VISIA scores between IPL and IPL + TA. No significant disparities were observed in VISIA-derived melanin parameters (Spots, UV Spots, Brown Spots, Red Areas) between the 2 cohorts of melasma at baseline, 1-mo, and 3-mo post-treatment intervals, “ns” represents no significant difference. IPL = intense pulsed light, TA = tranexamic acid.

No statistically significant disparities were observed in the VISIA-derived melanin indices, namely Spots, UV Spots, Brown Spots, as well as the vascular parameter red areas, between the 2 cohorts of melasma patients at baseline, the 1-month and 3-month post-treatment interval (Fig. 1, *P* >.05).

**Figure 2.**
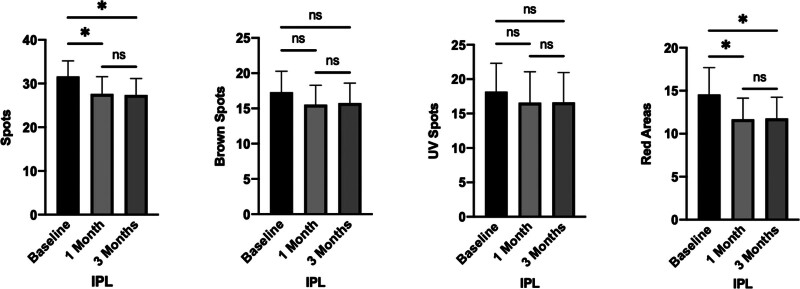
Comparison of VISIA scores before and after treatment in the IPL group. The scores for Spots and Red Areas exhibited statistically significant differences at both 1-mo and 3-mo post-treatment intervals, as compared to pretreatment levels (*P* <.05). In contrast, no significant differences were detected in the scores for UV Spots and Brown Spots at these time points when compared to baseline (*P* >.05). Additionally, there were no statistically significant disparities observed in the scores for all parameters (Spots, UV Spots, Brown Spots, Red Areas) when comparing the assessments at 1-mo and 3-mo post-treatment. The notation “ns” indicates *P* >.05, while * denotes *P* <.05. IPL = intense pulsed light.

Within the IPL group, a statistically significant difference was observed in the scores for both Spots and Red Areas at the 1-month and 3-month post-treatment intervals, as compared to baseline (*P* <.05 for both parameters). In contrast, no significant changes were detected in the scores for UV and Brown Spots at these time points relative to pretreatment levels (*P* >.05). Additionally, no statistically significant variations were observed in the scores for Spots, UV Spots, Brown Spots, and Red Areas when comparing the assessments at the 1-month and 3-month post-treatment. These findings are represented in Figure 2.

**Figure 3.**
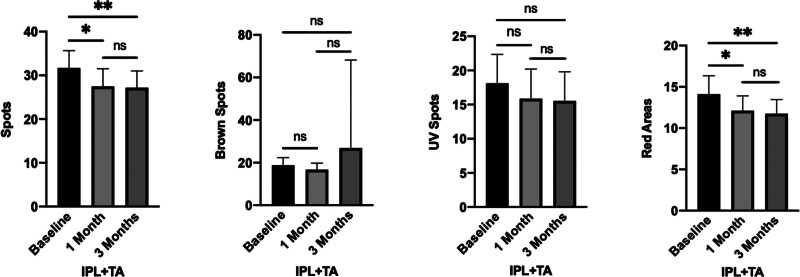
Comparison of VISIA scores before and after treatment in the IPL + TA group. At the 1-mo post-treatment mark, the scores for both Spots and Red Areas demonstrated statistically significant improvements compared to pretreatment levels (*P* <.05). These differences were further accentuated at the 3-mo post-treatment assessment (*P* <.01). Conversely, no statistically significant changes were observed in the scores for UV Spots and Brown Spots at either the 1-mo or 3-mo post-treatment time points when compared to baseline (*P* >.05). No significant differences were detected in the scores for all evaluated parameters (Spots, UV Spots, Brown Spots, Red Areas) when comparing the assessments at the 1-mo and 3-mo post-treatment. The notation “ns” denotes *P* >.05, * signifies *P* <.05, and ** indicates *P* <.01. IPL = intense pulsed light, TA = tranexamic acid.

Regarding the IPL + TA group, the scores for Spots and Red Areas showed statistically significant improvements at 1month post-treatmentcompared to baseline (*P* <.05), with even more marked differences observed at 3 months post-treatment (*P* <.01). However, no significant changes were observed in the UV Spots and Brown Spots scores at either 1 month or 3 months post-treatment relative to pretreatment levels (*P* >.05). Similarly, no statistically significant differences were noted in the scores for all parameters between the 1-month and 3-month post-treatment time points (Fig. 3).

**Figure 4.**
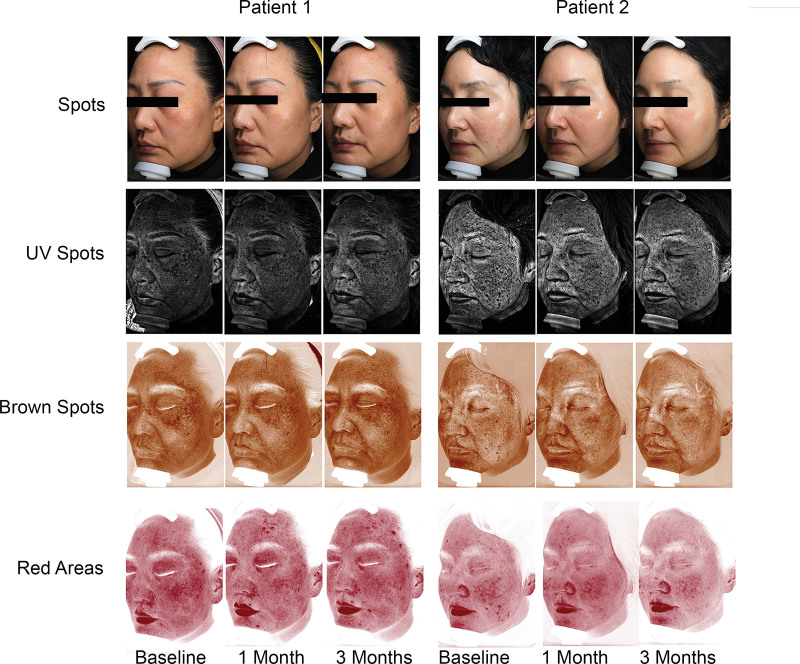
Representative images from VISIA. Patient 1 only received IPL treatment, while Patient 2 received IPL combined with TA treatment, IPL = intense pulsed light, TA = tranexamic acid.

Furthermore, Figure 4 presents a graphical illustration of the VISIA data for 2 melasma patients: Patient 1, who underwent IPL treatment, and Patient 2, who received IPL + TA therapy. The images depict the status of Spots, UV Spots, Brown Spots, and Red Areas at baseline, 1 month and 3 months following the final treatment session.

### 3.3. MASI score results

**Figure 5.**
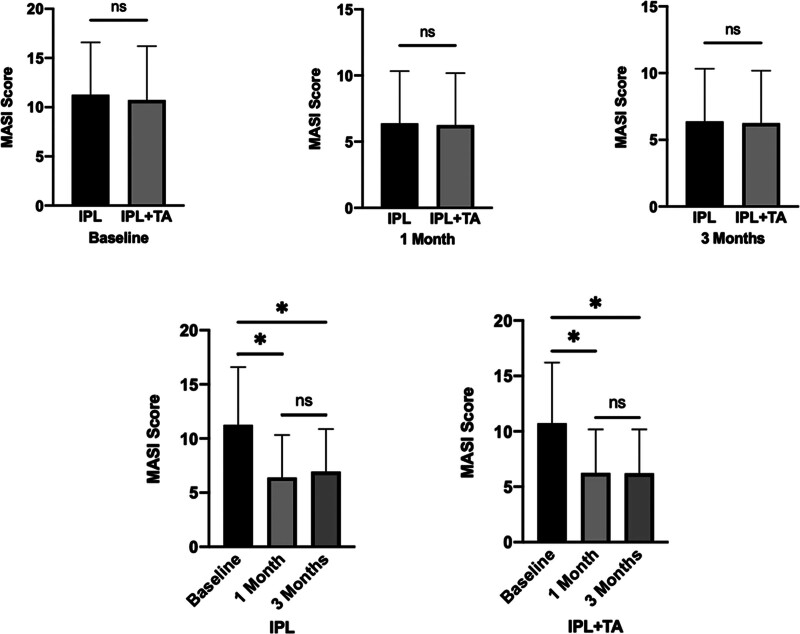
Comparative analysis of MASI scores before and after treatment in both groups. At baseline, there was no statistically significant difference in MASI scores between the 2 patient groups (*P* >.05). Similarly, no significant disparities in MASI scores were observed between the groups at the 1-mo and 3-mo post-treatment evaluations. However, within each group, there was a notable and statistically significant decrease in MASI scores at both the 1-mo and 3-mo follow-up assessments compared to their respective baseline scores (The notation “ns” denotes *P* >.05, * *P* <.05). IPL = intense pulsed light, MASI = Melasma Area and Severity Index, TA = tranexamic acid.

At baseline, no statistically significant difference was found in the MASI scores between IPL and IPL + TA (*P* >.05). Similarly, no significant differences in MASI scores were observed between the 2 groups at the 1- and 3-month post-treatment assessments (*P* >.05). However, the MASI scores at both 1 month and 3 months post-treatment were significantly lower than those at baseline (*P* <.05). Specifically, in the IPL group, the baseline MASI score of 11.3 ± 5.1 decreased significantly to 6.4 ± 3.8 at 1month post-treatment (*P* = .0167) and further improved to 6.9 ± 3.8 at 3 months post-treatment (*P* = .0374). In the IPL + TA group, the baseline MASI score of 10.7 ± 5.3, also showed a significant reduction to 6.2 ± 3.8 at 1month post-treatment (*P* = .0256) and remained stable at 6.2 ± 3.8 at 3 months post-treatment (*P* = .0242) (Fig. 5).

### 3.4. Patient satisfaction assessment

One month after the initiation of treatment, the satisfaction rate for the IPL group was recorded at 71.4%, whereas the IPL + TA group reported a higher satisfaction rate of 86.7%. At 3 months post-treatment mark, the satisfaction rate for the IPL group declined to 64.3%, while the IPL + TA group maintained a consistent satisfaction rate of 86.7%. IPL + TA group exhibited higher satisfaction rates compared to the IPL group at both the 1-month and 3-month post-treatment. Notably, the satisfaction rate in the IPL group decreased between the 1-month and 3-month follow-ups, whereas the satisfaction rate in the IPL + TA group remained stable (Table 3).

**Table 3:** Patient satisfaction assessment.

Group	Month	Dissatisfaction	Poor satisfaction	Satisfaction	Very satisfaction	Overall satisfaction rate (n)%
IPL	1	2	2	5	5	71.4
IPL + TA		1	1	6	7	86.7
IPL	3	4	1	4	5	64.3
IPL + TA		1	1	5	8	86.7

IPL = intense pulsed light, TA = tranexamic acid.

### 3.5. Recurrence

During the 3-month follow-up period, recurrence was observed in 3 patients in the IPL group, corresponding to a recurrence rate of 21.5%. Conversely, in the IPL + TA group, only one patient experienced a recurrence of skin lesions, resulting in a notably lower recurrence rate of 6.7% as compared to the IPL group.

### 3.6. Safety assessment

Twenty-nine patients with melasma completed 3 treatment sessions. Throughout the photorejuvenation process, neither the IPL group nor the IPL + TA group reported any significant pain or discomfort. Post-treatment, no adverse reactions such as blistering, pigmentation changes, or scarring were observed. In the IPL + TA group, all patients experienced a mild tingling sensation during the microneedle-assisted administration of TA, however, this pain was well-tolerated. Immediately following treatment, an erythematous reaction reaction occurred, but it resolved quickly with the application of cold compresses. Importantly, no adverse reactions, including infection, rash, redness, swelling, desquamation, scarring, pigmentation changes, or menstrual irregularities, were reported after the microneedle treatment.

## 4. Discussion

This study explores the effectiveness of combining IPL therapy with microneedling-assisted administration of TA for the treatment of melasma, and compares the results with those obtained with IPL therapy alone. According to both the VISIA assessment and the MASI scoring, no significant differences were found between the 2 groups. However, both treatment approaches yielded positive outcomes. Notably, at the 1-month and 3-month post-treatment follow-ups, the combination of IPL andTA showed more marked improvements in addressing Spots and Red Areas. Additionally, it is important to emphasize that the combined IPL + TA therapy not only enhances patient satisfaction but also diminishes the risk of recurrence. This study represents the first demonstration highlighting the unique benefits of this combined therapy in mitigating melasma-related pigmentation and vascular irregularities.

IPL stands out as a prominent physical therapy modality, widely used to address facial pigmentation and photoaging. By leveraging the principle of selective photothermolysis, IPL precisely targets melanin in the skin upon irradiation. This mechanism facilitates the formation of a thin epidermal crust that naturally sloughs off, while another fraction of melanin is efficiently cleared by phagocytic cells, ultimately achieving a spot-reduction effect. Furthermore, IPL’s ability to penetrate the epidermis and be absorbed by hemoglobin enables the coagulation and subsequent closure of blood vessels, effectively mitigating capillary dilation and addressing vascular conditions. Thus, IPL proves versatile in treating both pure melanized type (M type) and combined melanized with vascularized type (M + V type) melasma. Earlier IPL devices were characterized by sharp-peaked energy waves, where excessively high peak energies could result in skin burns and pigmentation issues. However, the IPL device used in this study incorporates advanced AOPT technology, offering unparalleled energy output and stability. This innovation allows for individual adjustment of each sub-pulse’s energy and pulse width, tailored to the specific lesion. Consequently, patients experience milder epidermal reactions, enhanced comfort, minimal post-treatment side effects, and accelerated recovery. The outcomes of this study are notably satisfactory, with no discernible adverse reactions reported during or after treatment. Previous research has corroborated the clinical efficacy of IPL in managing melasma,^[9,10]^ demonstrating its capacity to alleviate severity and boost patient satisfaction ratings. Nonetheless, certain studies have indicated a recurrence rate associated with IPL treatment for melasma,^[11]^ necessitating maintenance therapy to prevent recurrences or mitigate pigmentation reappearance. Therefore, there is a pressing need to explore novel clinical strategies to effectively control melasma recurrence. This study has achieved notably gratifying results through the synergistic application of IPL and TA.

TA, as a derivative of lysine, inhibits tyrosinase activity by specifically targeting and blocking the lysine binding sites on plasminogen molecules, effectively halting plasminogen activation. This mechanism subsequently mitigates the formation of new blood vessels and curtails the increase in melanin production induced by elevated arachidonic acid levels. Furthermore, TA reduces melanin synthesis by disrupting the paracrine signaling between mast cells, keratinocytes, and melanocytes.^[12]^ Although oral administration of TA has shown efficacy in treating melasma,^[13]^ concerns regarding long-term safety and contraindications for certain patients limit its widespread clinical use. Consequently, the therapeutic potential of topical TA for melasma has garnered considerable interest. However, the transdermal absorption of topically applied TA remains a challenge, restricting its clinical efficacy.^[14]^ To overcome this hurdle, microneedles have been employed to create micro-channels through the epidermis, facilitating drug delivery to the dermis. This approach not only reduces the required dosage but also enhances the drug’s effectiveness.^[15]^ Indeed, research has validated the safety and efficacy of microneedle-assisted delivery of TA for the treatment of melasma.^[13,16]^

Monotherapy, whether pharmacological or laser-based, often yields suboptimal results for the melasma treatment. Consequently, combined therapies have been adopted to enhance treatment outcomes.^[17]^ The findings of this study further corroborate the advantages of such combined approaches. Our observations indicate that while IPL monotherapy leads to sustained vascular improvement in melasma patients 3 months post-treatment, the improvement in pigmentation tends to decline, with a risk of recurrence. Conversely, the combination of IPL and microneedle-assisted TA delivery results in significant improvements in both pigmentation and vascular dilation at one and 3 month post-treatment, with effects persisting up to 3 months. Interestingly, no significant differences were observed in UV Spots and Brown Spots scores before and after treatment. This discrepancy may be attributed to the complex interplay of pigmentation at various depths within the skin, potentially obscuring changes in UV Spots and Brown Spots scores.^[5]^ Notably, patients treated with the combination therapy exhibited lower MASI scores, reduced recurrence rates, and higher overall treatment satisfaction compared to those treated with IPL alone. These findings align with previous research highlighting the synergistic effects of combined therapies.^[18]^ In conclusion, this study demonstrates that the combination of IPL and microneedle-assisted TA delivery is more effective than IPL monotherapy for the treatment of melasma, yielding higher patient satisfaction and a lower recurrence rate at 3 months post-treatment.

Taken together, this study demonstrates that both IPL monotherapy and the combination of IPL with TA delivery are viable treatment options for melasma. However, the combination therapy has proven to be superior, exhibiting enhanced clinical efficacy, improved recurrence rates, higher patient satisfaction, and minimal adverse effects. These attributes render it a safe and efficacious combined therapeutic approach for managing melasma. Nonetheless, this study has limitations. The sample size is relatively small, and there is no treatment group that received only microneedle-assisted TA delivery. Additionally, the follow-up duration was insufficient to fully assess the long-term effects of the treatments. To confirm and expand upon these findings, further research with larger sample sizes and extended follow-up periods is necessary.

## Author contributions

**Conceptualization:** Xin Mu.

**Data curation:** Rongli Yang, Yan Zhao, Jintian Luo.

**Formal analysis:** Rongli Yang.

**Investigation:** Rongli Yang, Chang Liu, Yiyang Bai.

**Methodology:** Xin Mu, Yan Zhao, Jintian Luo, Chang Liu, Yiyang Bai.

**Writing – original draft:** Rongli Yang.

**Writing – review & editing:** Xin Mu, Shaoli Cheng.
